# Harnessing skin-resident γδ T cells for immunotherapy in cutaneous squamous cell carcinoma

**DOI:** 10.1126/sciadv.aec7215

**Published:** 2026-06-24

**Authors:** Giorgia Nasi, Leonie C. Schöftner, Lara Ronacher, Amalia Sophianidis, Julia Feiser, Teodora Aleksandrova, Oliver Nussbaumer, Andrew Hutton, Roland Zauner, Johanna Moser-Waxenecker, Suraj R. Varkhande, Anshu Sharma, Monika Ettinger, Teresa Burner, Martin Laimer, Christina Guttmann-Gruber, Iris K. Gratz

**Affiliations:** ^1^ Department of Biosciences and Medical Biology, University of Salzburg, Salzburg, Austria.; ^2^ Institute of Medical Systems Biology, Johannes Kepler University Linz, Linz, Austria.; ^3^ GammaDelta Therapeutics Ltd, London, UK.; ^4^ Takeda Pharmaceuticals, Oncology Drug Discovery Unit, Cambridge, MA, USA.; ^5^ EB House Austria, Research Program for Molecular Therapy of Genodermatoses, Department of Dermatology and Allergology, University Hospital of the Paracelsus Medical University, Salzburg, Austria.; ^6^ Department of Dermatology and Venereology, Kepler University Hospital Linz, Linz, Austria.; ^7^ Department of Dermatology, Paracelsus Medical University Salzburg, Salzburg, Austria.; ^8^ Center for Tumor Biology and Immunology (CTBI), University of Salzburg, Salzburg, Austria.; ^9^ Benaroya Research Institute, Seattle, WA, USA.

## Abstract

Gamma delta (γδ) T cells are critical for tissue immune surveillance and their presence in tumors correlates with a favorable prognosis, highlighting their therapeutic potential. Although γδ T cells are abundant in the skin, their therapeutic value in skin cancer has remained largely unexplored due to challenges in isolating sufficient numbers of γδ T cells from human tissues and a lack of suitable preclinical models for skin cancer. Here, we are using innovative methods to expand human cutaneous γδ T cells ex vivo, enabling us to investigate their therapeutic potential in a human cutaneous squamous cell carcinoma (cSCC) mouse model. In this model, γδ T cells were specifically recruited to and maintained in the cSCC xenograft. These tumor-infiltrating cells exhibited an activated, cytotoxic phenotype and demonstrated effective antitumor activity in vivo. Collectively, our findings provide preclinical evidence supporting human skin–resident γδ T cells as a promising immunotherapeutic approach for treating skin cancers such as cSCC.

## Teaser

Human cutaneous γδ T cells infiltrate human cutaneous squamous cell carcinoma xenografts and exert potent antitumor effects.

## INTRODUCTION

γδ are unconventional T lymphocytes that play an important role in tissue immune surveillance (*1*, *2*). They are major histocompatibility complex (MHC) unrestricted, allowing their use in allogeneic cell therapy settings (*3*, *4*), and they express natural cytotoxicity receptors, such as natural killer protein 30 (NKp30), which recognize molecules up-regulated by stressed and malignant cells (*5*–*7*). γδ T cells can be rapidly activated and induce apoptosis of the target cells via several mechanisms, including the perforin-granzyme pathway, tumor necrosis factor (TNF)–related apoptosis-inducing ligand (TRAIL) and Fas ligand signaling or antibody-dependent cellular cytotoxicity (*8*–*11*). γδ T cells exhibit potent antitumor immunity against a wide range of solid and hematopoietic tumors (*12*–*14*), and tumor-infiltrating γδ T cells represent the main favorable prognostic immune cell population among 39 cancer types (*15*). Together, these features make γδ T cells ideal candidates for adoptive cancer immunotherapy.

On the basis of their δ chain expression, human γδ T cells can be subdivided into three main subsets, in which Vδ2^+^ are predominantly found in the blood whereas Vδ1^+^ and Vδ3^+^ are found in epithelial tissues (*16*, *17*). Vδ1^+^ γδ T cells account for 80 to 90% of the total γδ T cells skin and gut. These tissue-resident Vδ1^+^ γδ T cells have garnered attention as potential cancer therapeutics that have natural tissue tropism. Cutaneous squamous cell carcinoma (cSCC) is the second most common epithelial malignancy (*18*) and although most cases of cSCC are easily cured with surgery or ablation, a subset of these tumors recur, metastasize, and cause death (risk of approximately 2.1%) (*19*). In a mouse model of chemically induced cSCC, absence of γδ T cells correlated with increased tumor occurrence, which was not observed in mice lacking alpha beta (αβ) T cells, suggesting their important role in tissue surveillance (*20*). Although γδ T cells can infiltrate cSCC in patients (*21*), their therapeutic potential remains unexplored because up to now, the use of tissue-resident Vδ1^+^ γδ T cells in preclinical settings was limited because of the small numbers of cells recovered after tissue digestion. We studied the function of human skin–derived Vδ1^+^ γδ T cells expanded from healthy donor (HD) skin in a cSCC xenografting model. We have established a model that allowed us to analyze their migration, homing, and phenotypic adaptation in the human cSCC xenograft tissues. In this preclinical model, we found that ex vivo expanded cutaneous Vδ1^+^ γδ T cells controlled tumor growth. Overall, our results demonstrate the therapeutic efficacy of Vδ1^+^ γδ T cells in a mouse xenograft model supporting the strategy to use skin-resident Vδ1^+^ as an adoptive cell therapy against cutaneous malignancies.

## RESULTS

### Human cSCC xenografts release proinflammatory γδ T cell recruitment factors

**Fig. 1.**
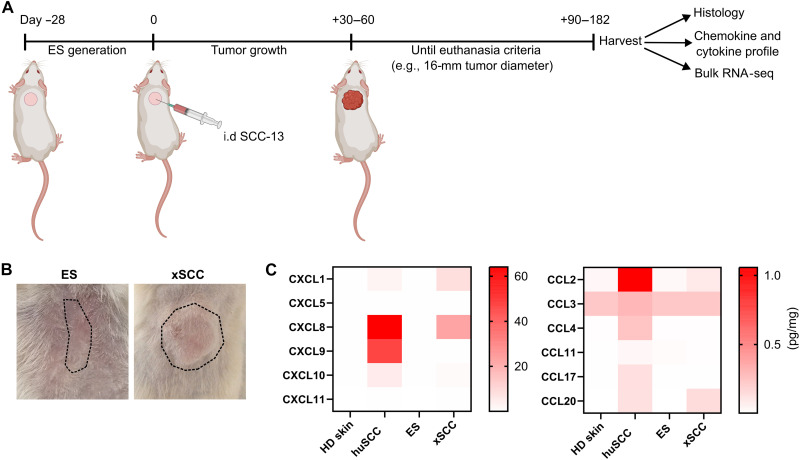
Xenograft cSCC produced proinflammatory Vδ1^+^ γδ T cell recruitment factors. (**A**) An in vivo cSCC xenograft mouse model (xSCC) was generated as following: first, engineered skin (ES) was formed on the back of NSG mice using a protocol established by Klicznik *et al.* (*22*). 1 × 10^6^ E6E7 immortalized human keratinocytes (huKCs) and human fibroblasts (huFibs) from a HD (HC-111) were transplanted into NSG mice. After complete healing of the wound, 0.3 × 10^6^ tumor cells (SCC-13) were i.d. injected into the newly generated ES. A palpable tumor bulge formed within 30 to 60 days postinjection and tumor size was measured twice per week until euthanasia criteria was reached. ES and xSCC were collected for subsequent analysis. Figure created in BioRender. I. Gratz (2026) https://BioRender.com/zmnd28b. RNA-seq, RNA sequencing. (**B**) Representative clinical picture of an ES and a xSCC. (**C**) Chemokine production by healthy human skin (HD skin), human cSCC (huSCC), ES and xSCC, expressed as pg/mg tissue, was measured by LEGENDplexTM. Heatmap represents the mean of *n* = 5 HD skin and huSCC donors and mean *n* = 5 of recipient mice.

To study the therapeutic potential of human cutaneous γδ T cell in human skin tumors, we first established an in vivo SCC xenograft mouse model (xSCC). To best match a human skin microenvironment, we first generated an engineered skin (ES) in which cSCC cells (SCC-13) were intradermally (i.d.) injected. In this method, cultured immortalized human keratinocytes (KCs) and fibroblasts (Fibs) isolated from human skin biopsies of an HD (HC-111) are placed in a grafting chamber that is surgically implanted on immunodeficient NSG mice. The KCs and Fibs undergo spontaneous cell sorting to form epidermal and dermal layers generating ES tissue with histological features of human skin as well as the organotypic expression of structural proteins such as human type VII collagen at the epidermal-dermal junction as previously described (*22*). After full differentiation of the ES (28 days) (Fig. 1, A and B), in vitro expanded SCC-13 cells, a spontaneous UV induced human SCC cell line (*23*, *24*), were i.d. injected into the ES. Palpable tumors were detectable between 30 to 60 days after injection, and tumors reached volumes of approximately 1300 mm^3^ after 90 to 182 days (Fig. 1, A and B, and fig. S1A). xSCC tumors displayed histological characteristics of SCC tumors in humans, specifically atypical epithelial cells with central keratinization and horn pearl formation (fig. S1B). Transcriptional analysis of xSCC versus ES and human SCC versus HD skin tissue revealed shared key transcriptional features, including metabolism (glycolysis and oxidative phosphorylation), DNA repair, and signaling pathways (e.g., interferon response) (fig. S2, A and B). Human SCC and xSCC had up-regulated expression of genes encoding two known γδ T cell ligands, UL16-binding protein 3 (ULBP3) and MHC class I chain–related protein A (MICA) (fig. S2C). Similarly, TNF receptor superfamily, member 10b, a TRAIL receptor, was up-regulated in both (fig. S2C). These molecules might promote tumor recognition by γδ T cell and enhance their ability to kill cSCC. In addition, on protein level, xSCC and human cSCC, but not ES and healthy human skin, shared a proinflammatory chemokine profile (Fig. 1C). Specifically, xSCC and SCC produced CXCL1, CXCL8, CXCL10, and CCL20 (Fig. 1C). Accordingly, human SCC and xSCC had up-regulated the expression of genes encoding CXCL1, CXCL8, and CXCL10 (fig. S2D). Together, the xSCC model shared key features with patient-derived tumors including their potential to recruit immune cells.

### Human cutaneous γδ T cells are recruited to and maintained in the tumor tissue

**Fig. 2.**
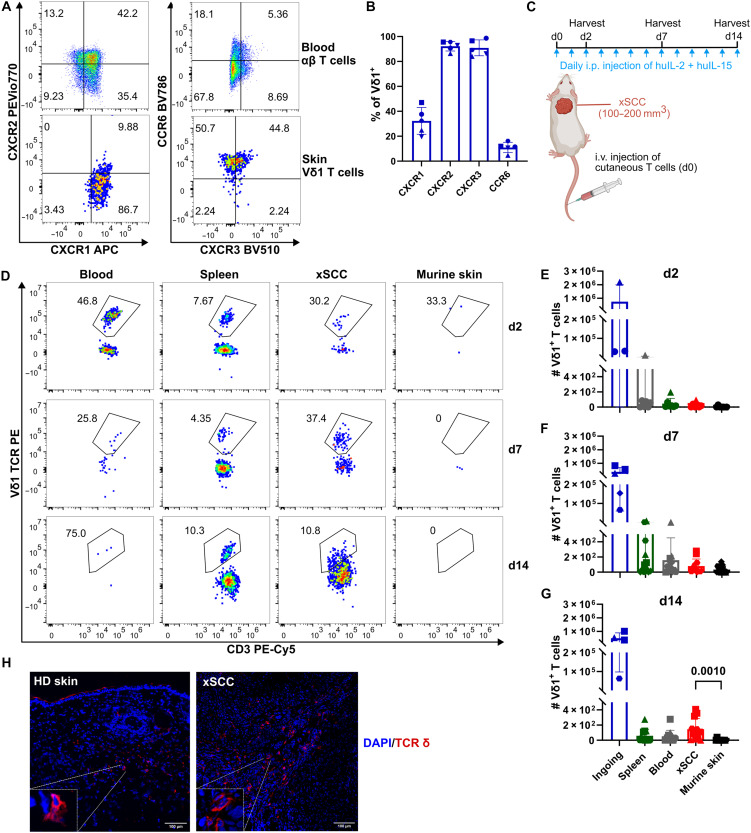
Human cutaneous Vδ1^+^ γδ T cells were maintained up to 14 days in xSCC. (**A**) Expression of indicated chemokine receptors by in vitro expanded, live gated CD3^+^ Vδ1^+^ γδ T cells. Blood-derived αβ T cells were used as staining control. (**B**) As (A), graphical summary of the percentage of Vδ1^+^ γδ T cell expressing the chemokine receptors (*n* = 5 skin donors). Error bars represent mean ± SD. (**C**) 10 × 10^6^ human skin–derived T cells, containing approximately 7% of Vδ1^+^ γδ T cells, were injected intravenously (i.v.) into NSG mice carrying a xSCC of a volume ranging from 100 to 200 mm^3^ [reached approximately 60 to 80 days (d) post–i.d. injection of SCC-13 cells]. Each mouse was injected intraperitoneally (i.p.) with recombinant IL-2 and IL-15 daily until the harvest day. Figure created in BioRender. I. Gratz (2026) https://BioRender.com/zmnd28b. (**D**) Representative plot of the percentage of Vδ1^+^ γδ T cells engrafted in the spleen, blood, xSCC and murine skin 2, 7, or 14 days posttransfer. (**E** to **G**) Bar graphs show the absolute numbers of ingoing Vδ1^+^ γδ T cells, Vδ1^+^ γδ T cells engrafting spleen and blood normalized to mouse weight (grams), and xSCC and murine skin normalized to tissue weight (grams). (E) *n* = 7 mice per group; pool of two independent experiments. (F) *n* = 12 mice per group; pool of four independent experiments; (G) *n* = 12 mice per group; pool of two independent experiments. Each symbol represents one skin donor. Error bars represent mean ± SD. Statistical significance was determined using the Kruskal-Wallis test with Dunn’s multiple comparisons test. All data points, including extreme values, are shown. (**H**) Representative immunofluorescent staining of colocalized TCRδ/DAPI in HD skin and xSCC 7 days after γδ transfer. Scale bars, 100 μm. Staining controls are shown in fig. S4.

On the basis of the production of chemokines by xSCC, we determined the chemokine receptor expression profile of human skin–derived Vδ1^+^ γδ T cells, which we sought to investigate using the xSCC model. Substantial fractions of Vδ1^+^ γδ T cells expressed CXCR1 and CXCR2, which are known receptors for CXCL1 and CXCL8 (*25*), and CXCR3, which binds the chemokine CXCL10 (*26*) (Fig. 2, A and B). A small fraction also expressed CCR6, which binds CCL20 (*27*) (Fig. 2, A and B). Collectively, these data suggest that Vδ1^+^ γδ T cells have the potential to respond to the proinflammatory chemokines released by the xSCC and to be actively recruited into the xSCC tissues. To directly test this, we adoptively transferred 10 × 10^6^ human skin–derived T lymphocytes (containing approximately 7% Vδ1^+^ γδ T cells and 93% αβ T cells), into NSG mice bearing xSCC at a volume ranging between 100 and 200 mm^3^. To maintain γδ T cell survival, we administered human interleukin-2 (IL-2) and human IL-15 daily by intraperitoneal (i.p.) injection until the end of the experiment (Fig. 2C). Vδ1^+^ γδ T cells, as well as αβ T cells, were already detectable in xSCC 2 days posttransfer and the number of Vδ1^+^ γδ T cells increased in xSCC over time (Fig. 2, D to G). Tumor-infiltrating γδ T cells were also identified using immunohistochemical analysis of xSCC (Fig. 2H and fig. S4). Vδ1^+^ γδ T cell numbers decreased over time in blood and spleen, in line with their migration into the xSCC tissue (Fig. 2, D to G). In line with these observations, there was a moderate positive correlation between the number of ingoing Vδ1^+^ and the number of Vδ1^+^ that engrafted in xSCC tissues, whereas no such relationship was observed in blood or spleen (fig. S3). Together, these data suggest that tumor accumulation is not merely due to systemic distribution but rather reflects preferential retention or survival within the tumor microenvironment. One xSCC bearing mouse developed a liver metastasis that contained γδ T cells 14 days posttransfer (fig. S5, A and B), which suggests that γδ T cells can infiltrate the primary and the metastatic tumor.

Since the ingoing population of T cells contained both, γδ and αβ T cells, we could monitor their relative engraftment in the xSCC and found that their ratio did not change over time (comparing days 2, 7, and 14), suggesting that both populations migrated and survived in the xSCC with similar efficacy (fig. S6, A and B). Little to no cells were detected in murine skin, suggesting that the systemic (i.p.) administered human cytokines alone were not sufficient to sustain γδ T cell survival (Fig. 2, D to G). Similarly, whereas initial engraftment of Vδ1^+^ γδ T cells in ES was observed, these cells were not maintained by day 14, suggesting that human skin alone may be insufficient to support their persistence (fig. S7A). This is consistent with the idea that the xSCC preferentially maintains or expands γδ T cells. Notably, αβ T cells displayed a similar pattern, as their numbers remained stable in ES, whereas they increased over time in xSCC (fig. S7B). Together, these findings suggest tissue-dependent differences in the maintenance and accumulation of transferred T lymphocyte subsets.

**Fig. 3.**
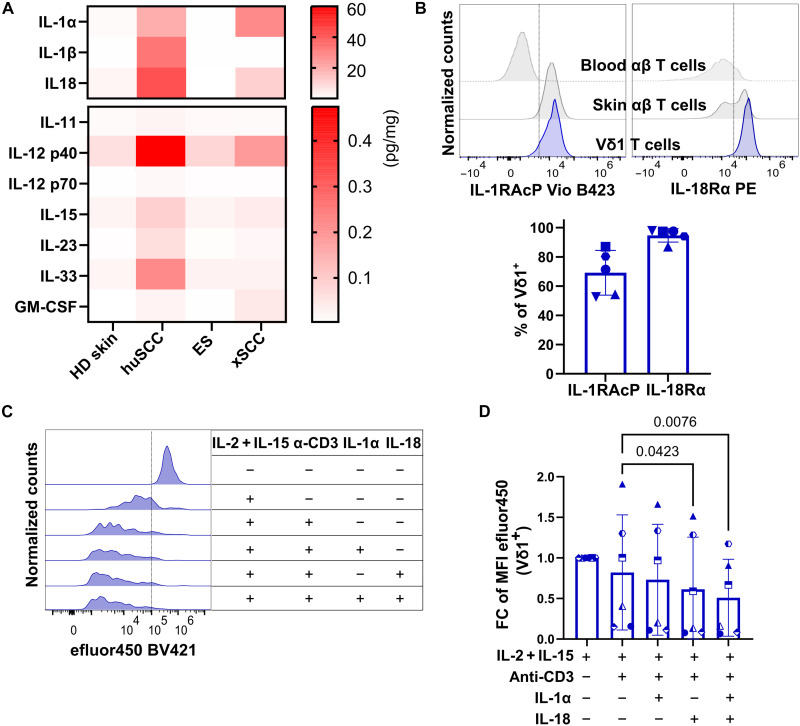
xSCC produced IL-1α and IL-18, which enhanced anti-CD3 induced-Vδ1^+^ γδ T cell proliferation in vitro. (**A**) Levels of cytokines (pg/mg tissue) produced by HD skin, huSCC, ES, and xSCC. Heatmap bars represent the mean of *n* = 5 HD skin and huSCC donors, and mean *n* = 5 of xenograft mice. (**B**) Representative gating strategy and bar graphs of the human skin–derived and ex vivo expanded Vδ1^+^ γδ T cells expressing IL-1RAcP and IL-18Rα. Peripheral blood αβ T cells and ex vivo expanded skin-derived αβ T cells were used as staining controls. Mean of *n* = 5 skin donors. (**C**) eFluor450-labeled γδ T cells were cultured under basal conditions [unstimulated or with IL-2 (100 IU/ml) and IL-15 (20 ng/ml)] or stimulated with anti-CD3 (1 μg/ml) and/or IL-1α and IL-18 (9 ng/ml) for 6 days. Proliferation was assessed by the median fluorescence intensity (MFI) of eFluor450 in Vδ1^+^ by flow cytometry. The representative histograms show the eFluor450 dilution in Vδ1^+^ T cells in the different conditions. Cell counts were normalized to unit area. (**D**) Bar graphs show the fold change of efluor450 MFI of Vδ1^+^ treated with anti-CD3, IL-1α, and IL-18 relative to IL-2 and IL-15. Mean of *n* = 6 skin donors. Statistical analysis was performed using a Friedman test followed by Dunn’s multiple comparisons test. Data in bar graphs (B) and (D) are shown as mean ± SD.

On the basis of the accumulation of the γδ T cells in the xSCC tissue, we sought to investigate whether the tumor microenvironment can support the proliferation of γδ T cells. We found that xSCC, similar to human SCC, produced the proinflammatory cytokines including IL-1α and IL-18, which might sustain cutaneous γδ T cell proliferation in the tissue (Fig. 3A) (*28*, *29*). The majority of Vδ1^+^ γδ T cells expressed the IL-1 receptor accessory protein (IL-1RAcP), a subunit that is essential for the effective signaling of IL-1 family cytokines, and IL-18 receptor α chain (IL-18Rα) (*30*) (Fig. 3B). The addition of IL-18 alone or the combination of IL-18 and IL-1α significantly enhanced the proliferation of γδ T cells when added to standard condition cultures in vitro (anti-CD3, IL-2, and IL-15) (Fig. 3, C and D). By contrast, IL-18 and IL-1α, in the absence of anti-CD3 stimulation, did not induce γδ T cell proliferation, indicating that T cell receptor (TCR)–mediated activation is required for these cytokines to exert their proliferative effect (fig. S8).

Collectively, these data showed that human cutaneous γδ and αβ T cells survived in vivo in the presence of IL-2 and IL-15, migrated to xSCC and were detectable in the tumors at least 14 days after the adoptive transfer of the cells. In addition, adoptively transferred γδ T cells may be maintained or even expanded within the xSCC cells in response to IL-1α and IL-18, which act together to enhance the proliferation of activated γδ T cells.

### Cutaneous tumor-resident γδ T cells exhibit an effector memory phenotype

**Fig. 4.**
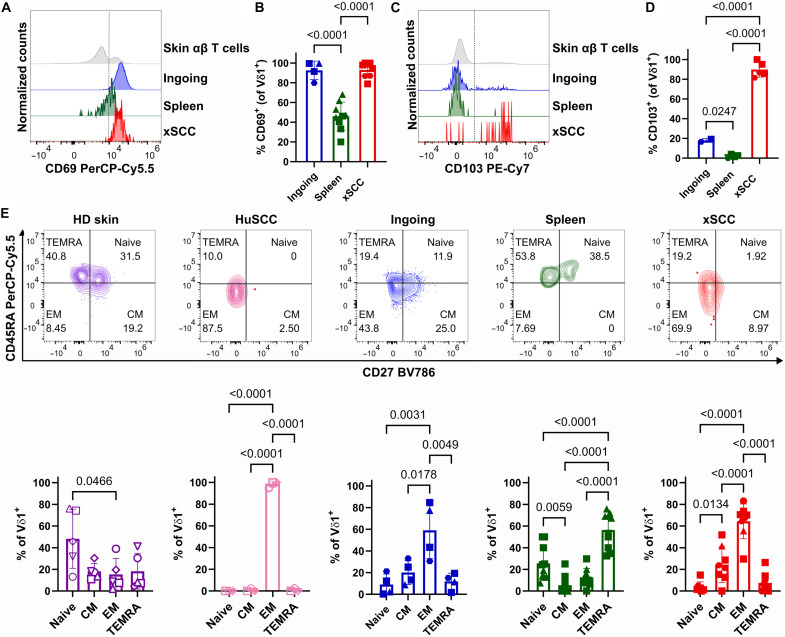
Vδ1^+^ γδ T cells from xSCC tissues expressed tissue retention markers and displayed a T_EM_ phenotype. (**A** and **C**) Representative flow cytometry histograms of CD69 and CD103 expression by human skin–derived and ex vivo expanded Vδ1^+^ γδ T cells pretransfer (ingoing) and isolated 14 days posttransfer from spleen and xSCC. Human skin–derived and ex vivo expanded αβ T cells were used as staining controls (gray histograms). Cell counts were normalized to unit area. (**B** and **D**) Bar graphs show the percentage of Vδ1^+^ γδ T cell expressing CD69 and CD103 in ingoing Vδ1^+^, and Vδ1^+^ isolated 14 days posttransfer from spleen and xSCC. (B) *n* = 15 mice per group, pool of five independent experiments. (D) *n* = 8 to 9 mice per group, pool of two independent experiments. (**E**) Representative FACS plots and graphical summary of the proportion of naïve, central memory (CM), effector memory (EM), and late effector memory (TEMRA) in Vδ1^+^ γδ T from HD skin (*n* = 5), huSCC (*n* = 3), ingoing Vδ1^+^ (*n* = 4) and Vδ1^+^ from spleen (*n* = 10 mice, pool of two independent experiments) and xSCC (*n* = 8 mice, pool of two independent experiments) 14 days after i.v. injection. Each symbol represents one skin donor. Error bars are shown as mean ± SD. Data were analyzed using ordinary one-way ANOVA with Tukey’s multiple comparisons test.

On the basis of the notion that the tumor cells secreted molecules that affected γδ T cells, we next aimed to elucidate phenotypical adaptations of transferred γδ T cells upon infiltration of the xSCC tissue. We found that the majority of γδ T cells within the xSCC expressed key markers associated with tissue residency, CD69 and CD103 (Fig. 4, A to D). Ingoing Vδ1^+^ γδ T cells were CD69^hi^, but approximately 50% of cells lost CD69 in the spleen but not in xSCC. (Fig. 4, A and B, and fig. S9A). The proportion of CD103^+^ Vδ1^+^ γδ T cells was similar between spleen and xSCC after 7 days from γδ T cell injection (fig. S9B). However, after 14 days, approximately 80% of Vδ1^+^ within the xSCC were CD103 positive, whereas only a small percentage of CD103^+^ γδ T cells were detectable in the spleen (Fig. 4, C and D). Overall, these data are consistent with the idea that tumor-infiltrating γδ T cells assumed tissue residency, with CD69 and CD103 acting to promote their retention in the SCC tissue and to limit their recirculation, similar to αβ^+^ tissue-resident memory (T_RM_) populations (*31*, *32*). Thus, we next investigated the proportion of the memory subpopulations within the tumor-infiltrating γδ T cells, using the coexpression of CD27 and CD45RA. We found that the majority of γδ T cells isolated either from SCC of patients or SCC xenografts displayed a CD45RA^−^CD27^−^ effector memory phenotype (Fig. 4E) (*33*, *34*). These data suggested that cutaneous γδ T cells can assume a tissue-resident effector memory phenotype within the human tumor tissue but not murine tissues implying direct cellular communication with the tumor cells.

### Cutaneous γδ T cells isolated from xSCC display an activated and cytotoxic phenotype

**Fig. 5.**
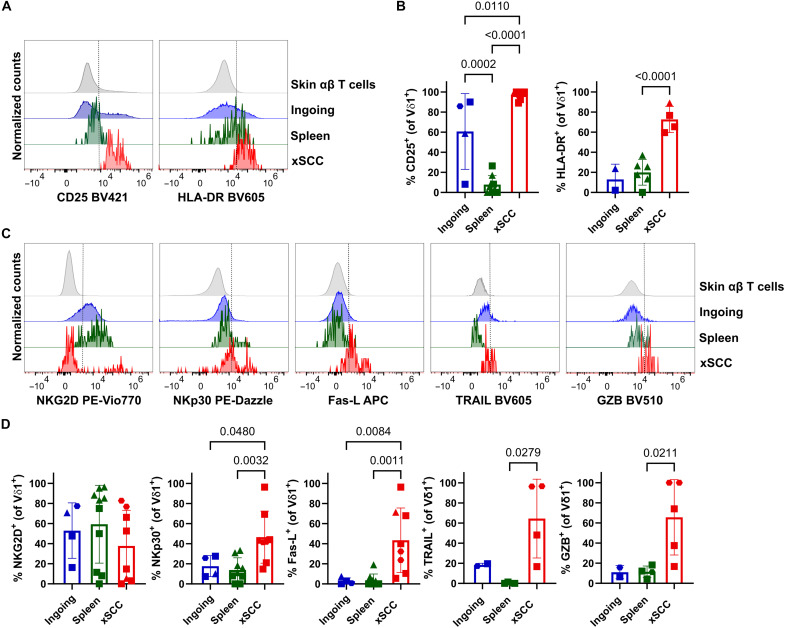
Vδ1^+^ γδ T cells isolated from xSCC tissues expressed activation and cytotoxic markers. (**A** and **B**) Representative FACS histograms and graph bars showing the percentage of human skin–derived and ex vivo expanded Vδ1^+^ γδ T cell expressing CD25 (*n* = 8 to 9 mice per group, pool of two independent experiments), and HLA-DR (*n* = 4 to 6 mice per group, one experiment) before injection and 14 days posttransfer in spleen and xSCC. (**C** and **D**) Representative FACS plots and fraction of Vδ1^+^ γδ T cells expressing NKG2D (*n* = 8 to 10 mice per group, pool of two independent experiments), NKp30 (*n* = 8 to 9 mice per group, pool of two independent experiments), Fas-L (*n* = 8 to 9 mice per group, pool of two independent experiments), TRAIL (*n* = 4 to 5 mice per group, one experiment), and granzyme B (GZB) (*n* = 4 to 5 mice per group, one experiment) in the ingoing population, spleen and xSCC 14 days after i.v. injection. Human skin–derived and ex vivo expanded αβ T cells were used as staining controls (gray histograms). Histograms were normalized to unit area. Each symbol represents one skin donor. Error bars represent mean ± SD. Data were analyzed using ordinary one-way ANOVA with Tukey’s multiple comparisons test.

In addition to assuming a tissue-resident phenotype, we observed that the frequencies of CD25^+^ and HLA-DR^+^ γδ T cells were increased in xSCC tissues when compared to γδ T cells pretransfer and γδ T cells from the spleen, suggesting γδ T cell activation in the tumor tissues (Fig. 5, A and B, and fig. S9, C and D). Moreover, increased fractions of xSCC tumor-infiltrating γδ T cells expressed several cytotoxic markers, such as NKp30, Fas-L, TRAIL, and granzyme B, 14 days posttransfer when compared to the spleen (Fig. 5, C and D). This cytotoxic phenotype is consistent with that observed in xSCC metastasis, although this is based on a single sample (fig. S5, A and B). Increased frequency of NKp30^+^ γδ T cells was seen as early as 7 days after transfer, in line with the findings from Correia *et al.* (*35*), where blood-derived Vδ1^+^ γδ T cells up-regulated NKp30 within the first week of TCR and cytokine stimulation (fig. S9F). In contrast, the expressions of Fas-L and TRAIL after 7 days posttransfer remained unchanged, suggesting a different kinetic (fig. S9, G and H). Only a small fraction of ingoing γδ T cells expressed NKp30 and Fas-L, which implies that the tumor microenvironment promoted the expression of these two markers among Vδ1^+^ γδ T cells (Fig. 5, C and D). Fold change analysis of median fluorescence intensity (MFI) was overall consistent with the frequency data, in line with tissue-dependent modulation of HLA-DR, Fas-L, TRAIL, and granzyme B (fig. S10, A to D). Moreover, Vδ1^+^ γδ T cells maintained their expression of activating immunoreceptor natural killer group 2, D (NKG2D) as ingoing cells (approximately 40 to 60%) (Fig. 5D and fig. S9E). Overall, xSCC-infiltrating Vδ1^+^ γδ T cells displayed an activated and cytotoxic phenotype.

### Expanded cutaneous γδ T cells confer an antitumor response against xSCC

**Fig. 6.**
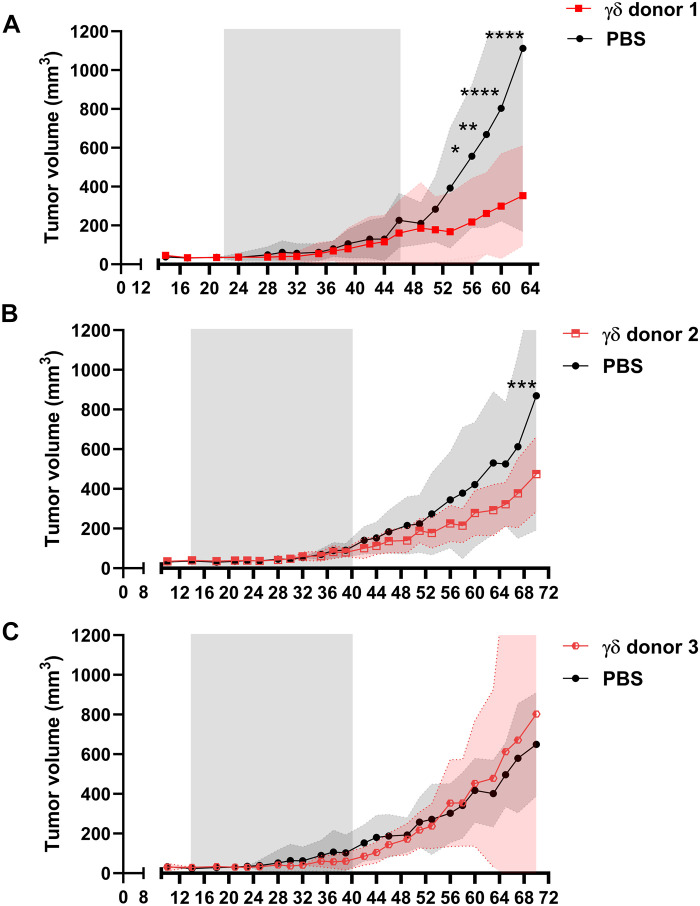
Antitumor response of in vitro expanded skin-derived γδ T cells. (**A** to **C**) Cutaneous Vδ1^+^ γδ T cells were ex vivo expanded and γδ T cells subsequently isolated by magnetic cell sorting. 4 × 10^6^ purified γδ T cells (>90%) from three skin donors or PBS were injected intravenously in mice when the tumor reached a volume between 30 and 50 mm^3^. HuIL-2 and huIL-15 were i.p. injected every day (gray area) for (A) 25 days, or (B and C) for 26 days. Tumor volume was determined from measurements conducted three times per week using a caliper. (A) *n* = 4, (B) *n* = 6, (C) and *n* = 5 mice per group, one experiment. Solid line shows the mean response; shaded area indicates 95% CI across replicates. Data were analyzed using two-way ANOVA with Šídák’s multiple comparisons test.

Last, to investigate the antitumor efficacy of human skin–resident γδ T cells against cSCC cells, one dose of in vitro expanded and purified γδ T cells (purity >90%; fig. S11) from three different HD skins were transferred into xSCC bearing mice. Control xSCC bearing mice were injected with phosphate-buffered saline (PBS). Human recombinant IL-2 and IL-15 were administered in both groups for 25 to 26 days posttransfer. Notably, γδ T cells from two of three healthy skin donors reduced tumor growth compared to the PBS group (Fig. 6, A to C).

To directly compare the cytotoxic capacity of cutaneous γδ T cells and cutaneous αβ T cells, we performed an in vitro luminescence-based killing assay using AkaLuc-expressing SCC cells as targets. At a low effector-to-target ratio (2:1), cutaneous γδ T cells exhibited increased tumor cell killing compared with αβ T cells, although this difference did not reach statistical significance (fig. S12). At higher effector-to-target ratios (5:1 and 10:1), both cutaneous γδ and αβ T cells displayed comparable cytotoxic activity (fig. S12). These results indicate that while both populations are capable of effective tumor cell killing in vitro, cutaneous γδ T cells may exhibit enhanced activity at low effector-to-target ratios with the added advantage of them not being alloreactive. Overall, these data provide the first evidence that skin-resident γδ T cells can be used in immune therapy for cutaneous cancers.

## DISCUSSION

**Fig. 7.**
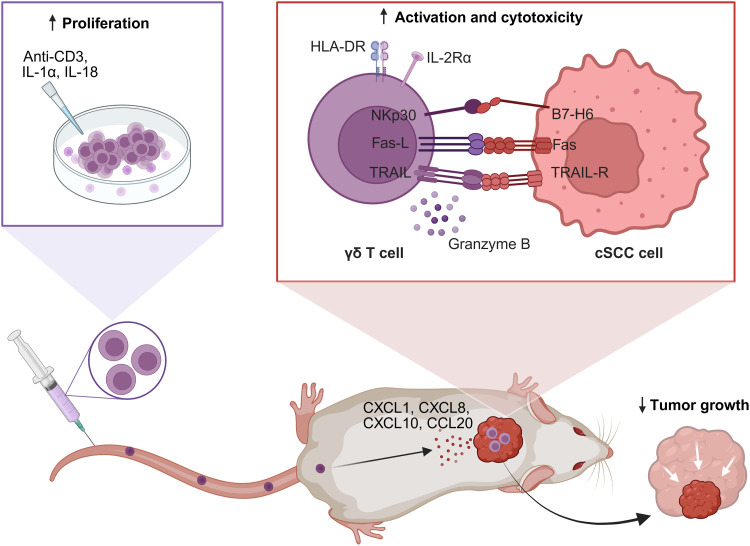
Graphical summary of the main findings of the manuscript. Human cutaneous γδ T cells, isolated from skin biopsies of HDs and adoptively transferred into recipient mice, infiltrated the human cSCC xenograft from the circulation, homed into the tumor tissue, and were maintained within the tumor. γδ T cells can proliferate following TCR activation and stimulation with IL-1α and IL-18, which are also produced by the tumor tissue. cSCC-infiltrating γδ T cells displayed an activated and cytotoxic phenotype, and infiltration can lead to reduced tumor growth. Figure created in BioRender. I. Gratz (2026) https://BioRender.com/m9vgwti.

γδ T cells have features that make them ideal candidates for cancer immunotherapy, such as the recognition of stress-induced antigens expressed on the surface of malignant cells, potent cytotoxicity against a broad range of tumor cell types and TCR independence from MHC recognition, which allows their use in allogeneic therapies. Over the past decade several clinical trials have been carried out using predominantly blood derived Vδ2^+^ γδ T cells, which showed no side effects but varying clinical efficacy between patients (*3*, *36*). Compared to Vδ2^+^, Vδ1^+^ γδ T cells constitute the major population in the epithelial tissues and have a natural tissue tropism, which can facilitate their migration and infiltration into solid tumors (*37*, *38*). Moreover, a study evaluated clinical responses in patients with stage IV melanoma treated with adoptive transfer of autologous tumor-infiltrating lymphocytes containing Vδ1^+^ T cells (*39*). One patient, who received the highest number of Vδ1^+^ γδ T cells, achieved complete remission (*39*). The infused Vδ1^+^ cells were safe and well tolerated; however, because of the presence of other cell types, such as CD8^+^ T cells, the specific therapeutic role of Vδ1^+^ cells could not be determined. Despite the clinical relevance and ssubstantial global mortality of cSCC (*19*), no clinical studies on cSCC have been performed so far. Preclinical studies with suitable humanized mouse models do not currently exist, hindering investigation of human Vδ1 γδ T cell tissue biology. In the present study, we described the phenotypic adaptation and clinical efficacy of human skin–resident γδ T cells in an in vivo humanized cSCC mouse model. A graphical summary of the main findings of this manuscript is shown in Fig. 7.

We demonstrated that human skin–resident γδ T cells from HDs expressed chemokine receptors conducive to the migration into the SCC xenograft. Adoptively transferred γδ T cells were selectively maintained in the tumor tissue, as well as in the tumor metastasis, introducing the idea that human cutaneous γδ T cells might be used for the treatment of primary as well as metastatic skin tumors. Although human IL-2 and IL-15 are crucial cytokines for γδ T cell survival, they were not sufficient to maintain Vδ1^+^ γδ T cells in spleen, blood, murine skin, and ES, suggesting that in addition to these cytokines, the tumor microenvironment was necessary to sustain γδ T cell persistence in this in vivo setting. For instance, the human epithelial cells of the SCC xenograft might provide trans-presentation of IL-15 to γδ T cells, which is required to achieve more robust signaling as compared to IL-15 alone (*40*). In addition, the tumor microenvironment might directly affect the action of these cytokines by secretion of IL-1α and IL-18. IL-1α is a potent inflammatory cytokine with the ability to induce indirect proliferation of T cells by up-regulating IL-2R expression (*28*). Moreover, the addition of IL-18 to circulating Vδ2^+^ γδ T cells, expanded in presence of zoledronate and IL-2, enhanced their proliferation (*41*). Unlike Vδ2^+^ γδ T cells, circulating Vδ1^+^ γδ T cells were largely unresponsive to the IL-18 treatment, in line with our findings (*33*). Notably, cutaneous Vδ1^+^ γδ T cells acquired increased sensitivity to IL-18 or IL-18 and IL-1α–induced proliferation upon TCR activation, a process that may preferentially occur within the tumor microenvironment. This context-dependent responsiveness suggests that tumor-derived cytokines could selectively support in situ expansion of transferred γδ T cells, representing a potential therapeutic advantage by promoting γδ T cell persistence within tumors. The dependency of γδ T cell survival to the constant administration of IL-2 and IL-15, however, poses some limitations in long-term preclinical mouse studies, which might partially be overcome by replacing the administration of recombinant IL-15 by using of NSG-Tg(Hu-IL15) mice, which express physiological levels of human IL-15 (*42*).

A high fraction of Vδ1^+^ isolated from SCC xenograft were activated and expressed markers of cytotoxic function, which suggests that the tumor microenvironment actively stimulated γδ T cells, driving them toward potent cytotoxic effector cells. We found that the xSCC xenografts expressed the γδ T cell ligands MICA and ULBP3, which is consistent with a contribution of NKG2D to SCC cell recognition and γδ T cell activation (*43*–*45*). Moreover, xSCC expressed TRAIL-R, highlighting TRAIL as a potential candidate for inducing cSCC cell death (*46*). In line with the observed increased expression cytotoxicity markers, γδ T cells demonstrated in vitro cytotoxic activity against SCC cells and showed a trend toward enhanced killing compared with cutaneous αβ T cells at low effector-to-target ratios, consistent with their rapid, innate tumor recognition capacity (*1*). At higher ratios, the comparable cytotoxicity of γδ and αβ T cells may reflect increased T cell activation strength or potential alloreactive or nonspecific effects under high effector conditions. Extending these findings in vivo, administration of γδ T cells isolated from two of three donors resulted in reduced tumor growth. This finding, combined with the activated and cytotoxic phenotype of tumor-infiltrating γδ T cells, provides preclinical evidence for the potential of these cells as a therapeutic strategy for cSCC. However, the fact that one γδ T cell donor did not show any response poses the need for follow-up studies to identify biomarkers that could predict the treatment response of a certain batch of γδ T cells and thus guide donor selection. The promising results of this study suggest that innate mechanisms of tumor recognition by Vδ1 can be sufficient for the antitumor response. These mechanisms might be further boosted by gene engineering of ingoing Vδ1^+^ γδ T cells, which could add further antitumor functionality to γδ T cells. Such engineering could include the expression of chimeric antigen receptors that target solid tumor antigens. Overall, our study provides early evidence that off-the-shelf, allogeneic human skin–resident γδ T cells can be used as a treatment option for patients with cSCC. It further demonstrates the usefulness of the established xenograft model to elucidate mechanisms underlying the observed differences in antitumor efficacy of individual γδ T cell batches and to better select or enhance the therapeutic cells.

## MATERIALS AND METHODS

### Study design

The objective of this preclinical study was to evaluate the migratory behavior, phenotype, proliferation, and antitumor efficacy of adoptive γδ T cell therapy. This was accomplished using γδ T cells that were isolated from human skin from HDs, expanded, stimulated in vitro, or adoptively transferred into mice that carried engineered human cSCC xenografts followed by flow cytometric analysis of their cellular phenotype and function. For γδ T cell migration and phenotypic characterization, data were collected at three predefined posttreatment points (days 2, 7, and 14). In the therapeutic efficacy studies, tumor-bearing mice (xSCC) were assigned to treatment groups in matched pairs based on similar tumor volumes and maintained in the same cage, to minimize baseline variability before treatment intervention. Blinding was not performed during treatment administration; however, tumor growth was assessed using objective measurements (e.g., caliper-based tumor volume) to reduce observer bias. Experiments were terminated when the first mouse within a cohort reached the humane endpoint, as defined by the approved animal protocol. To account for biological variability, adoptive γδ T cell products were derived from two to four healthy human donors (i.e., biological replicates) per experimental question. Experimental replicates were defined as independent in vivo experiments, comprising a minimum of four mice (range: 4 to 15) that had all received the same human material. This design ensured technical reproducibility across experiments and biological variability across donors.

### Mice

NOD-scid IL2Rgamma^null^ mice (NSG, RRID:IMSR_JAX:005557) were obtained from The Jackson Laboratory and bred and maintained in a specific pathogen–free facility in accordance with the guidelines of the Central Animal Facility of the University of Salzburg (*47*). Mice were housed in individually ventilated cages (≤5 per cage), with a 12-hour light/dark cycle, controlled temperature (21° to 23°C) and humidity (45 to 65%). Standard chow and water were available ad libitum, and environmental enrichment was provided. For the therapeutic experiments, only female mice were used. Animals were 20 ± 5 weeks old at the start of the experiment. For phenotypic assessments, both male and female mice were included. Sex was recorded and considered in the analysis where appropriate. Animals were 39 ± 5 weeks old at the start of γδ T cell injection. All animal procedures were approved by the Austrian Federal Ministry of Education, Science and Research under the protocol numbers 66-012/0036-WF/V/3b/2014 and 2025-0.714.862 and were conducted in accordance with institutional and national guidelines for animal welfare.

### Human tissue samples

Human skin samples, used for the expansion of skin-resident γδ T cells, were obtained via third party commercial suppliers (BioIVT and Tissue Solutions), which individually hold ethical approval for the patient consent and collection of healthy human skin samples from cosmetic surgery, and were derived from female donors, aged between 18 and 59 undergoing either abdominoplasty or breast reduction surgery. Human SCC biopsies were collected at the Department of Dermatology and Venereology, Johannes Kepler University of Linz in collaboration with the EB Biobank of the EB House Austria, Department of Dermatology and Allergology, University Hospital Salzburg, Austria, and were surgically removed from female and male patients, aged between 72 and 96 and clinically classified as grade G2. Human skin biopsies were provided by the Breast Center of the University Hospital Salzburg, and were derived from female donors, aged between 23 and 67 undergoing breast reduction surgery. All samples were obtained upon written informed consent. This study complies with all relevant ethical regulations and the use of the human samples for research approved by the ethics committee of the State of Salzburg, Austria. The study was conducted in accordance with the Helsinki Declaration principles.

### Cell lines and xSCC generation

Human KCs and Fibs were provided by EB House Austria and were isolated from human skin and immortalized using human papilloma viral oncogenes E6/E7 HPV as previously shown (*48*, *49*). Human cSCC cells (SCC-13, RRID:CVCL 4029) were provided by J. G. Rheinwald (University of California, Los Angeles, United States) and were isolated from a facial skin SCC and expanded in vitro as previously described (*24*). SCC-13 cells were stably transduced to express the monomeric red fluorescent protein mRuby (note: the fluorescence was not used in the analysis presented here). Human KCs and SCC cells were cultured in the CnT-Prime Epithelial Proliferation Medium (CELLnTEC, catalog no. CnT-PR) supplemented with primocin (50 μg/ml; InvivoGen, catalog no. ant-pm-1) and human Fibs were cultured in the CnT-Prime Fibroblast Proliferation Medium (CELLnTEC, catalog no. CnT-PR-F) containing primocin (50 μg/ml; InvivoGen, catalog no. ant-pm-1).

ES was generated as previously described (*22*, *50*). In brief, 1 × 10^6^ KCs and 1 × 10^6^ autologous Fibs were immortalized (*48*), expanded, and added to a silicone chamber implanted on the backs of NSG mice. Mice were anesthetized with isofluorane during the procedure and received buprenorphine (0.08 mg/kg s.c.) for analgesia. After at least 30 days to allow full differentiation of the ES, 3 × 10^5^ SCC-13 cells were i.d. injected into ES. Tumor size was measured with a caliper two to three times per week, and volume (mm^3^) was estimated with the ellipsoid approximation formula: width^2^ × length/2 (*51*). Mice were monitored daily for signs of pain and distress. Criteria for euthanasia included weight loss of more than 20% and a tumor size of 16 mm in diameter.

### Histology and immunohistochemistry

Punch biopsies (4 mm) of xSCC, human healthy skin (HD skin), and SCC tissues (huSCC) were fixed in 4% formaldehyde (ROTI Histofix, Carl Roth, catalog no. P087.5) for 24 hours, dehydrated, and embedded in paraffin according to standard protocols (*52*). Four-micrometer tissue sections were cut on a microtome (HistoCore AUTOCUT, Leica), mounted on SuperFrost Plus glass slides (Carl Roth, catalog no. H867.1), and deparaffinized in xylol (Carl Roth, catalog no. 9713.1) and descending ethanol (≥99.8%, Carl Roth, catalog no. K928.1) in H_2_O series. The sections were stained with iron hematoxylin (Sigma-Aldrich, catalog no. 1.04302) for 6 min and eosin (Carl Roth, catalog no. X883.1) for 2.5 min, dehydrated in ethanol and xylol, and sealed with synthetic resin. For immunofluorescence staining, HD skin and xSCC tissue were cryosectioned at 6 μm onto SuperfrostPlus Adhesion Slides (Epredia, catalog no. J1800AMNZ), fixed with cold acetone (Carl Roth, catalog no. 7328.1), blocked with 5% bovine serum albumin (Sigma-Aldrich, catalog no. A7030) for 1 hour and incubated with anti-human TCRδ (1:100, Santa Cruz Biotechnology, catalog no. sc-100289, RRID:AB_1130061) for 2 hours. Sections were washed and incubated with Alexa Fluor 647–conjugated secondary antibody (1:500, Thermo Fisher Scientific, catalog no. A-31571, RRID:AB_162542) for 1 hour, mounted with ProLong Diamond Antifade Mountant with DAPI (Invitrogen, catalog no. P36966) and imaged on a Nikon Ti2 microscope.

### Bulk RNA sequencing

Total RNA was extracted from biopsies of HD skin, huSCC, ES, and xSCC using the Monarch Total RNA Miniprep Kit (NEB, catalog no. TS2010). Samples were sent to the Biomedical Sequencing Facility at CeMM Research Center for Molecular Medicine of the Austrian Academy of Sciences for next generation sequencing. RNA quantity and integrity were assessed with Qubit 2.0 (Thermo Fisher Scientific) and Agilent 2100 Bioanalyzer (RNA 6000 Pico Kit, catalog no. 5067-1513). Libraries were prepared with the NEBNext Poly(A) mRNA Magnetic Isolation Module, the Ultra II Directional RNA kit, and Multiplex Oligos for Illumina (New England Biolabs). Library concentrations were quantified with Qubit 2.0 assay and library quality and fragment size distribution were assessed using the Agilent 2100 Bioanalyzer (High Sensitivity DNA Kit, catalog no. 5067-4626). Sample-specific libraries were pooled equimolarly and sequenced on NovaSeq 6000 (50 bp, paired-end) (Illumina, San Diego, CA, USA). Reads were mapped to the Genome Reference Consortium GRCh38/GRCm39 using STAR (version 2.7.11b) with GENCODE version 46/M35 annotations (*53*, *54*). Further downstream analysis was conducted using statistical software R (version 4.5.0). Low-count genes were filtered and trimmed mean of *M* values normalized with edgeR (version 4.6.2) (*55*). Differential gene expression (DEG) analysis was performed using limma/voom (version 3.64.1) (*56*). Genes with an adjusted (Benjamini & Hochberg) *P* value <0.01 and fold change >2-fold were considered significantly differentially expressed. Volcano plots and box plots were generated using ggplot2 (version 3.5.2) and ggpubr (version 0.6.0) (*57*). In volcano plots, top 20 based on adjusted *P* value <0.05 and fold change >2-fold of common up and down-regulated DEGs were highlighted. Common DEGs between huSCC versus HD skin and xSCC versus ES were used for gene set enrichment analysis against Molecular Signature Database (MSigDB) Cancer Hallmarks (*Homo sapiens*) using fgsea (10,000 permutations). A heatmap of concordantly enriched pathways was generated using pheatmap (version 1.0.12).

### Adoptive transfer of human skin–derived γδ T cells

Human skin–derived T lymphocytes, isolated from healthy human donor skins, were provided by GammaDelta Therapeutic and cultured in γδ T cells expansion media composed of cTexMacs medium, containing TexMacs medium (Miltenyi Biotec, catalog no. 130-097-196), 10% plasma derived pooled human serum (One Lambda, catalog no. A25761) and 1% penicillin/streptomycin (Sigma-Aldrich, catalog no. P0781), supplemented with a proprietary recombinant human cytokine cocktail to support Vδ1^+^ γδ T cell survival and proliferation. For phenotypic characterization, human skin–derived T lymphocytes, containing a fraction of Vδ1^+^ γδ T cells (stated in the respective legends), were cultured in presence of limited cytokine support and injected into SCC-bearing mice intravenously when tumors reached a volume between 100 and 200 mm^3^. rhIL-2 (0.5 μg per mouse, BioLegend, catalog no. 589108) and rhIL-15 (1 μg per mouse, Miltenyi Biotec, catalog no. 130-095-766) were i.p. injected daily for 7 or 14 days. To test therapeutic efficacy, human skin–derived T lymphocytes derived from skin of three different HDs were further expanded in vitro in γδ T cell expansion media, to generate a large pool of Vδ1^+^-enriched cells. Subsequently, in vitro expanded γδ T cells were isolated by magnetic cell sorting via positive selection, using biotin labeled anti-human TCRγδ antibody (Miltenyi Biotec, catalog no. 130-113-502, AB_2733575) to obtain highly purified γδ T cells (>90%). Pairs of mice were chosen on the basis of the similar tumor size (30 to 50 mm^3^) and randomly assigned to either the γδ or PBS control transfer group. In the γδ transfer group, 4 × 10^6^ purified γδ T cells were intravenously injected into SCC-bearing mice. In the control group, SCC-bearing mice were intravenously injected with an equal volume of PBS. rhIL-2 (0.5 μg per mouse, BioLegend, catalog no. 589108) and rhIL-15 (1 μg per mouse, Miltenyi Biotec, catalog no. 130-095-766) were i.p. injected daily for a maximum of 25 to 26 days.

### Tissue processing and flow cytometry analysis

To analyze γδ T cells after in vivo adoptive transfer, animals were euthanized using CO_2_ asphyxiation followed by cervical dislocation. Blood was collected using a Heparin Vacutainer (Greiner Bio-One, catalog no. 455084) from the heart and diluted in PBS in a 1:1 ratio. To obtain single-cell suspension, spleens were homogenized, the cell slurry filtered through a 70-μm cell strainers and red blood cells were lysed using BD Pharm Lyse Lysing Buffer solution (BD Biosciences, catalog no. 555899). Whole xSCC, huSCC, HD skin biopsies, and healthy liver biopsies were minced with dissection scissors and digested overnight in 5% CO_2_ at 37°C using collagenase type 4 (5.3 mg/g tissue, Worthington, catalog no. LS004188) and deoxyribonuclease I (DNase I) (200 U/ml, Sigma-Aldrich, catalog no. D5319) in cTexMacs medium. Four-millimeter punch biopsies of shaved dorsal murine skin were minced with dissection scissors and digested at 37°C for 45 min using collagenase from *Clostridium histolyticum* (2.0 mg/ml; Sigma-Aldrich, catalog no. C9407), hyaluronidase (0.5 mg/ml; Sigma-Aldrich, catalog no. H3506), and DNase (0.1 mg/ml; Sigma-Aldrich, catalog no. DN25-1G) as previously described (*58*). Single cells were filtered through 70-μm cell strainers, washed with PBS, and stained for flow cytometry (*31*). Used antibodies are listed in table S1. For intracellular staining, cells were incubated with brefeldin A (10 μg/ml; Sigma-Aldrich, catalog no. B7651) for 3 hours at 37°C and 5% CO_2_. Cells were permeabilized and fixed using a Cytofix/Cytoperm kit (BD Bioscience, catalog no. 554714). Data were acquired on a Cytoflex LS flow cytometer (Beckman Coulter), and data analyzed using FlowJo (FlowJo LLC, version 10.10).

### Proliferation assay

Human skin–derived T lymphocytes were labeled with Cell Proliferation Dye eFluor 450 (10 μM, Thermo Fisher Scientific, catalog no. 65- 0842-85), washed with PBS, and subsequently stimulated for 6 days with rhuIL-2 (100 IU/ml; BioLegend, catalog no. 589108), rhuIL-15 (20 ng/ml; BioLegend, catalog no. 570603), in addition with mouse anti-human CD3 monoclonal antibody (1 μg/ml; Miltenyi Biotec, catalog no. 130-093-387, RRID:AB_1036144) alone or with rhuIL-1α (9 ng/ml; BioLegend, catalog no. 570004) and rhuIL-18 (9 ng/ml, BioLegend, catalog no. 592102) applied individually or in combination in cTexMacs medium. Data were acquired on a Cytoflex LS flow cytometer (Beckman Coulter) and analyzed using FlowJo (FlowJo LLC, version 10.10). Proliferative activity of cells was determined from the MFI of eFluor 450 of the Vδ1 population.

### Multiplex chemokine and cytokine analysis

Punch biopsies (4 to 8 mm) of HD skin, ES, huSCC, and xSCC were homogenized in a protease inhibitor cocktail (Sigma-Aldrich, catalog no. P8340) at a concentration of 64 to 128 mg/ml using the SpeedMill Plus (Analytik Jena) and subsequently filtered using 0.22-μm Spin-X columns (Sigma-Aldrich, catalog no. CLS8161). Tissue homogenates were subjected to a bead-based multiplex LEGENDplex analysis, following the manufacturer’s instructions. Human proinflammatory chemokines, including C-X-C motif chemokine ligands (CXCL) IL-8 (CXCL8), IP-10 (CXCL10), MIG (CXCL9), ENA-78 (CXCL5), GROα (CXCL1), I-TAC (CXCL11), and CC chemokine ligands (CCL) eotaxin (CCL11), TARC (CCL17), MCP-1 (CCL2), RANTES (CCL5), MIP-1α (CCL3), MIP-3α (CCL20), and MIP-1β (CCL4) IP-10 (CXCL10) were quantified using a LEGENDplex Human Proinflammatory Chemokine Panel 1 kit (13-plex kit, BioLegend catalog no. 740985, RRID:AB_3683628). Human cytokines, including IL-1α, IL-1β, IL-11, IL-12p40, IL-12p70, IL-15, IL-18, IL-23, IL-27, IL-33, interferon-α2, TSLP, and granulocyte-macrophage colony-stimulating factor were quantified using LEGENDplex Human Cytokine Panel 2 kit (13-plex kit, BioLegend, catalog no. 741378). Reactions were performed in duplicates. Data were acquired using a Cytoflex LS flow cytometer (Beckman Coulter) and analyzed with Legendplex version 8.0 software (BioLegend).

### In vitro cytotoxicity assay

Tumor target cells (SCC-13) stably expressing the luminescent reporter AkaLuc were provided by F. Aberger and P. Krenn (Department of Biosciences and Medical Biology, University of Salzburg, Austria) and were used to assess cytotoxic activity. Target cells were seeded in 96-well white plates at a density of 8 × 10^3^ cells per well and allowed to adhere overnight. Human cutaneous γδ and αβ T cells from 3 HDs were in vitro expanded with γδ T cells expansion media and isolated by magnetic cell sorting via negative selection, using biotin labeled anti-human TCRαβ antibody (Miltenyi Biotec, catalog no. 130-133-896) or biotin labeled anti-human TCRγδ antibody (Miltenyi Biotec, catalog no. 130-113-502, AB_2733575), respectively. Cells purity was ≥90%. Purified γδ T cells or αβ T cells were added at effector-to-target ratios of 2:1, 5:1 and 10:1. Cocultures were maintained in the expansion media for 22 hours at 37°C in 5% CO_2_. Following incubation, luminescence was measured using a TECAN plate reader Infinite 200 PRO (Tecan Life Sciences) after addition of the AkaLuc substrate according to the manufacturer’s instructions. Luminescent signal intensity, expressed as relative light units, was used as a surrogate for viable target cell number. Percent cytotoxicity was calculated relative to target-only wells, which were defined as 0% killing.

### Statistical analysis

Analyses were performed in GraphPad Prism version 10.4 (GraphPad Software). To compare engraftment and phenotypic changes of γδ T cells across three independent groups (ingoing cells, spleen, and xSCC), data were first tested for normal Gaussian distribution. Non-Gaussian data were analyzed using a Friedman test or a Kruskal-Wallis *H* test and post hoc pairwise comparisons were performed with Dunn’s test corrected for multiple comparisons. Normally distributed data were analyzed using a one-way analysis of variance (ANOVA) to compare engraftment and phenotypic changes of γδ T cells across groups. Post hoc pairwise comparisons were performed with Tukey’s multiple comparisons test. Error bars indicate means ± SD. *P* < 0.05 was considered statistically significant. Tumor volume was measured at multiple time points and summarized using means and 95% confidence intervals (CIs).
