# Supplementary material for: Harnessing skin-resident γδ T cells for immunotherapy in cutaneous squamous cell carcinoma
Source: Sci Adv. 2026 Jun 24;12(26):eaec7215. doi: 10.1126/sciadv.aec7215 (PMC13292952; doi:10.1126/sciadv.aec7215)
Supplement: Supplementary file 1 — Figs. S1 to S12 Table S1 [file sciadv.aec7215_sm.pdf]

Science Advances
AAAS

Supplementary Materials for

Harnessing skin-resident γδ T cells for immunotherapy in cutaneous squamous cell carcinoma

Giorgia Nasi *et al.*

Corresponding author: Giorgia Nasi, giorgia.nasi@plus.ac.at

Sci. Adv. **12**, eaec7215 (2026)
DOI: 10.1126/sciadv.aec7215

This PDF file includes:

Figs. S1 to S12
Table S1

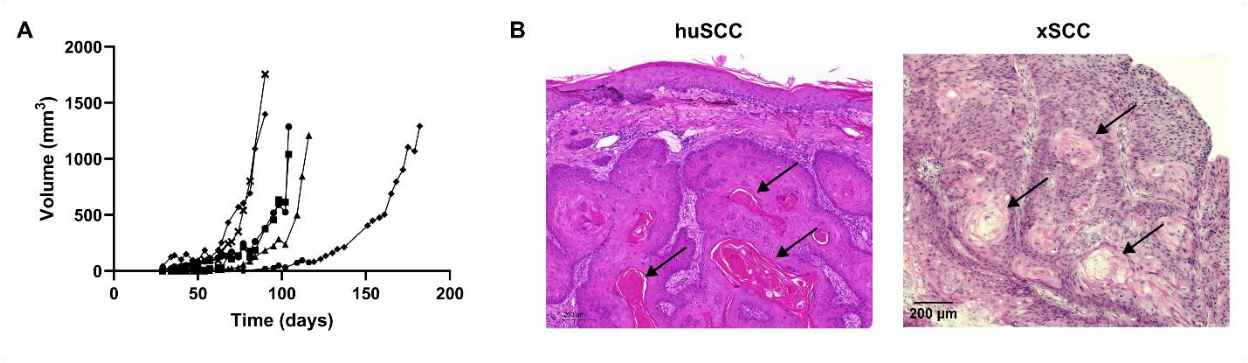

Figure S1. cSCC xenograft resemble features of cSCC of patients. (A, B) 0.3×10^6 tumor cells (SCC-13) were intradermally injected in engineered skin (ES) generated on the back of NSG mice. After 26 days, a palpable bulge was formed, and mice were monitored until euthanasia criteria was reached. (16 mm of tumor diameter). (A) Growth curve of xSCC, measured until euthanasia criteria were reached. Data are representative of n=6 recipient mice. (B) H&E staining of human cSCC (huSCC) classified at stage G1, compared to cSCC xenograft (xSCC) tissues harvested 116 days after SCC cell injection. Black scale bar = 200 µm. Arrows indicate the horn pearl formation.

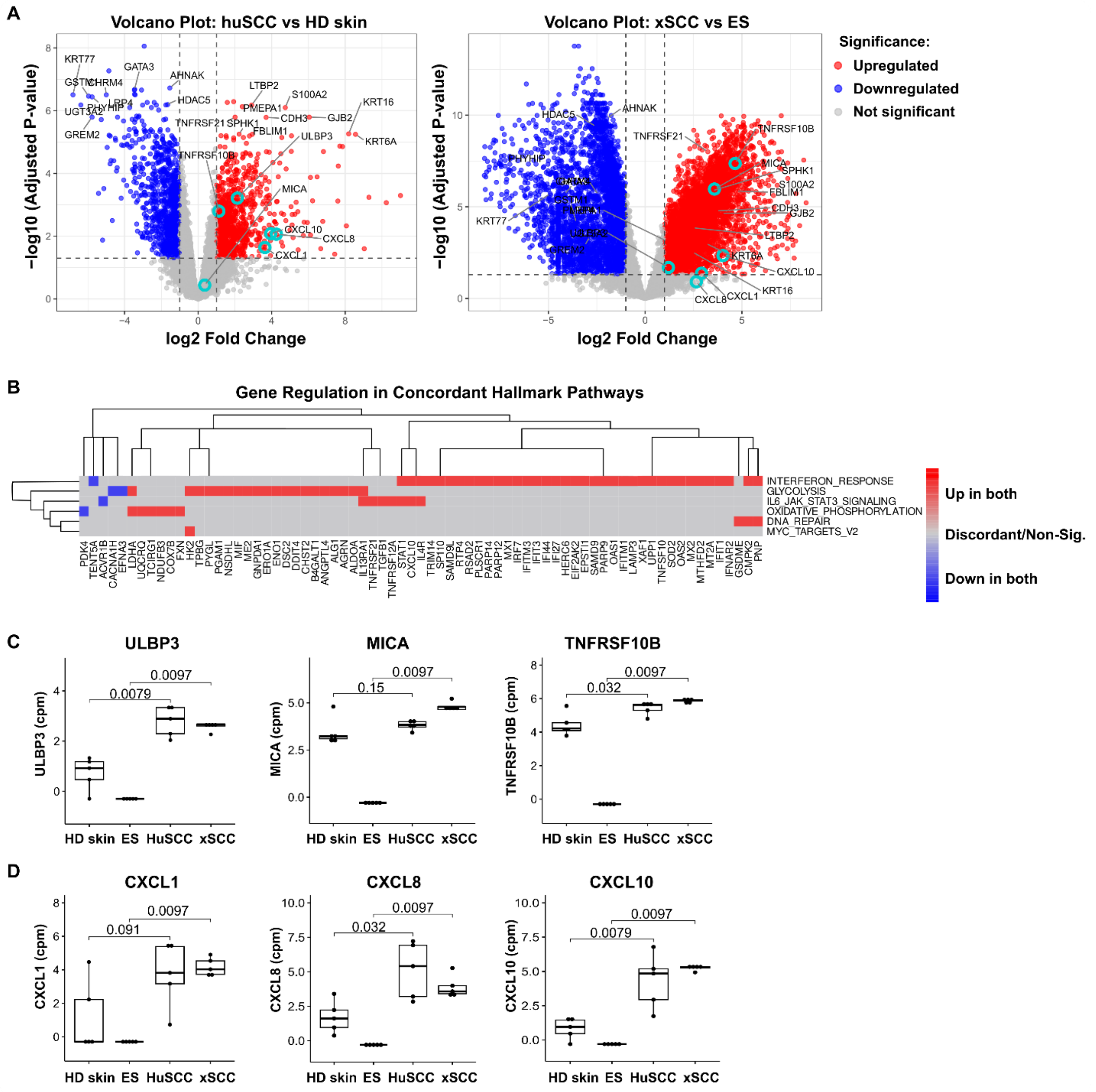

Figure S2. Transcriptomic signatures of cSCC xenografts. (A) Volcano plots showing 2-fold significantly (adjusted p-value < 0.05) up- (red), and down-regulated (blue) differentially expressed genes (DEGs) comparing huSCC vs HD skin (left graph) and xSCC vs ES (right graph). Top 20 genes sorted by adjusted p-value and foldchange (huSCC vs HD skin) of common deregulated genes (intersect) between human and xenograft are labelled. Relevant genes (ULPB3, MICA, TNFRSF10B, CXCL1, CXCL8 and CXCL10) to this study are highlighted in light blue.

(**B**) Heatmap shows significantly enriched (adjusted p-value < 0.05) concordant Molecular Signature Database cancer hallmark genesets (rows) between the human (HuSCC vs HD skin) and xenograft (xSCC vs ES) and genes which are concordantly (blue: down-, red: up-regulated) expressed in both models. (**C, D**) Boxplots showing the log2 transformed expression levels (counts per million, cpm) of UL16 binding protein 3 (ULBP3), MHC Class I Polypeptide-Related Sequence A (MICA) TNF Receptor Superfamily Member 10b (TNFRSF10B), CXCL1, CXCL8 and CXCL10 across HD skin, ES, huSCC and xSCC. Each dot in the boxplot represents one sample. Statistical analysis was performed using non-parametric Wilcox test.

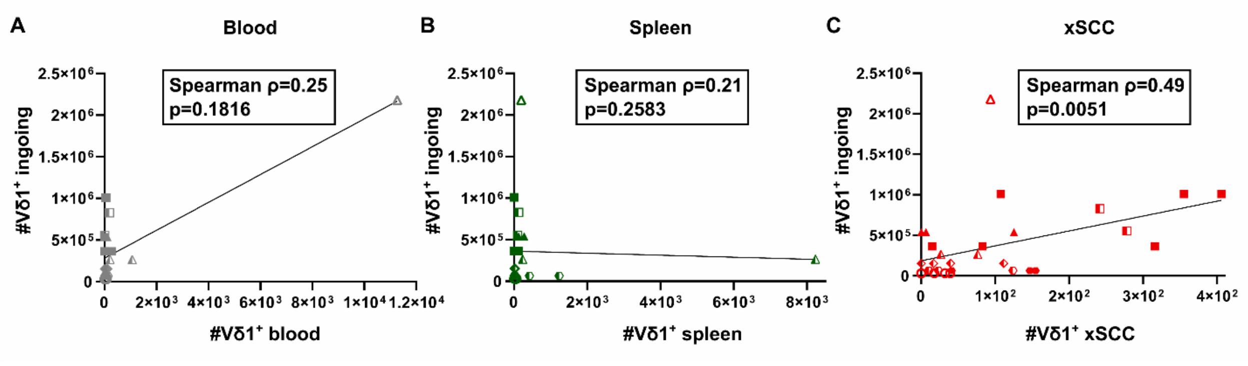

Figure S3. Correlation between ingoing Vδ1$^+$ T cells and Vδ1$^+$ T cells after engraftment across tissues. (A-C) Scatter plots show correlations between the absolute number of ingoing Vδ1$^+$ and the absolute number of Vδ1$^+$ engrafting spleen, blood and xSCC. Each symbol represents a γδ T cell donor. Time points are indicated by symbol fill: empty = day 2, half-filled = day 7, full = day 14. Solid lines indicate linear regression. Spearman rank correlation coefficients (ρ) and p-values are indicated in each panel.

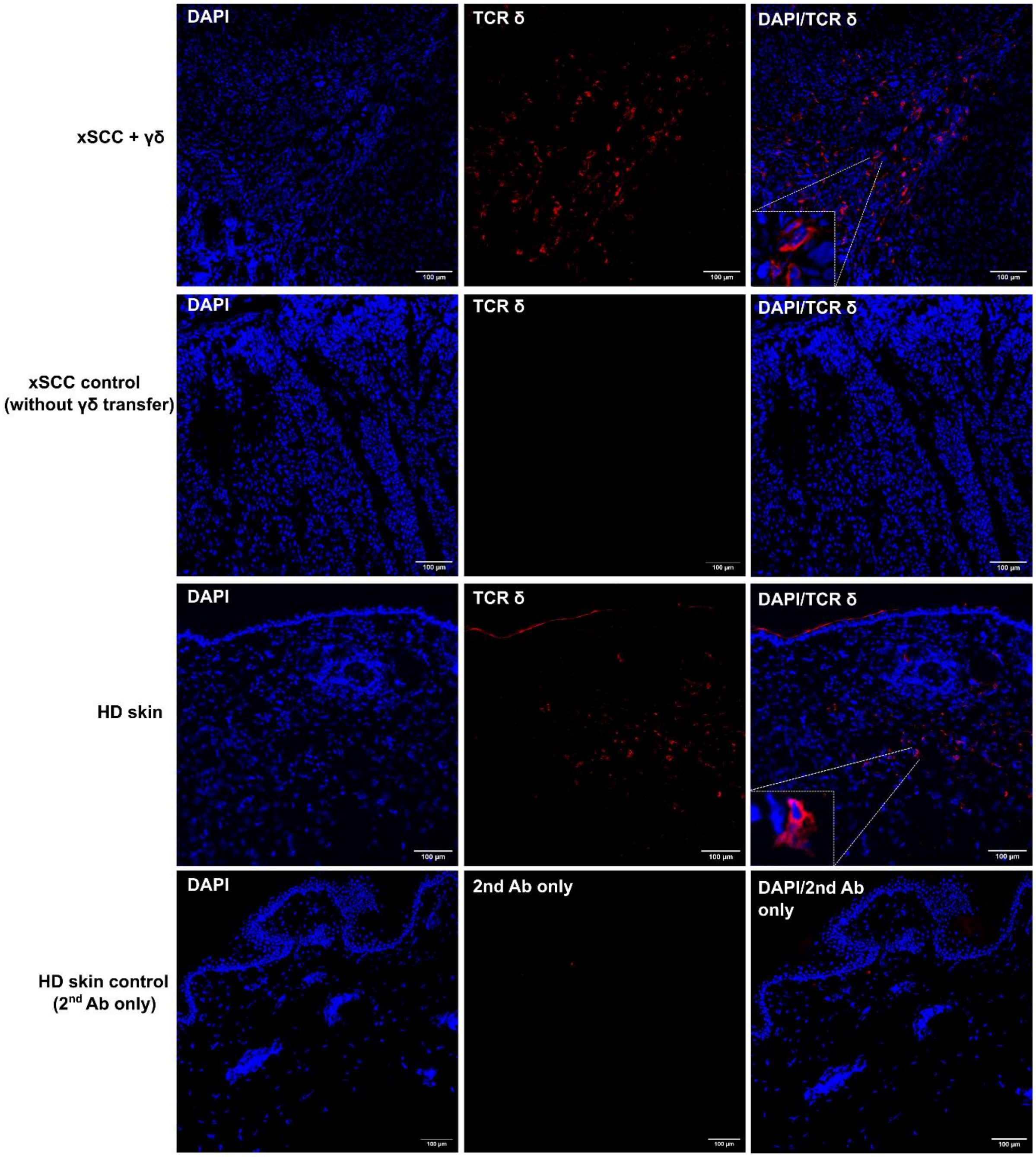

Figure S4. Skin resident γδ T cells infiltrated xSCC tissue. Representative immunofluorescent staining of DAPI (blue), TCR δ (red) and co-localized TCR δ /DAPI in HD skin and xSCC 7 days after γδ transfer. Scale bar 100 mm. xSCC control refers to xSCC without γδ T cell engraftment; HD skin control refers to HD skin without primary antibody (2nd Ab only).

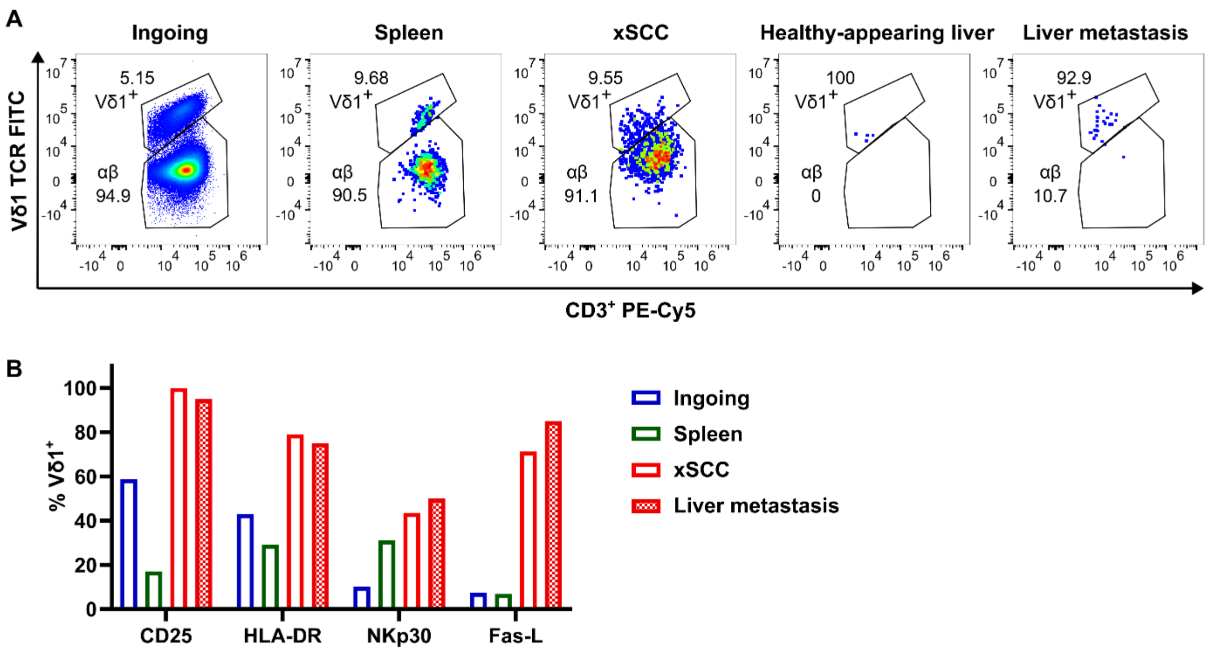

Figure S5. Human cutaneous γδ T cells engraft cSCC liver metastasis and showed similar phenotypic profile compared to γδ infiltrating the primary tumor. (A, B) $10x10^6$ cutaneous $CD3^+$ T cells, containing 4.65% of γδ T cells, were intravenously injected into a NSG mouse carrying a xSCC of a volume of 513 mm^3. Human recombinant IL-2 and IL-15 were daily injected until the harvest day (day 14). Spleen, xSCC, healthy-appearing liver, and liver metastasis were collected for flow cytometry analysis. (A) Representative gating strategy of the percentage of $V\delta1^+$ γδ and αβ T cells of $CD3^+$ cells in the ingoing population, spleen, xSCC, healthy-appearing liver and liver metastasis. (B) Summary graph showing the percentage of $V\delta1^+$ γδ T cells expressing CD25, HLA-DR, NKp30 and Fas-L in the ingoing population, spleen, xSCC and liver metastasis. Data are representative of one mouse.

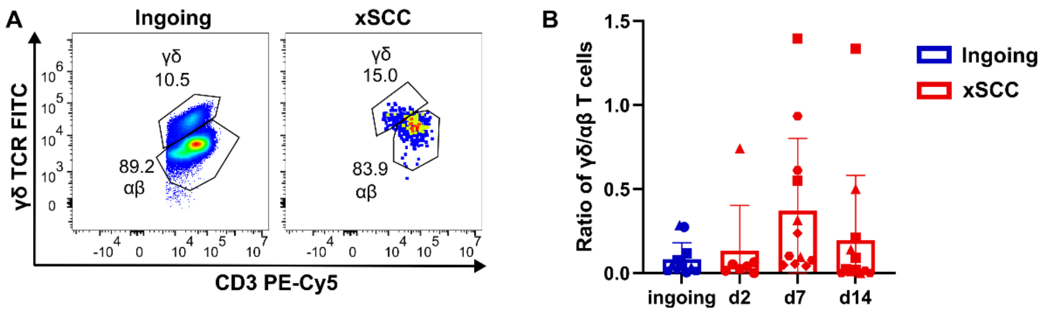

Figure S6. Proportion between γδ and αβ T cells in CD3$^+$ T cell population prior transfer was maintained in xSCC tissues. (A, B) 10x10^6 cutaneous CD3$^+$ T cells, with γδ T cells accounting for 7% ± 7,45, were intravenously injected into NSG mice carrying a xSCC of a volume ranging from 100 to 200 mm^3. Each mouse was intraperitoneally injected with recombinant IL-2 and IL-15 daily until the harvest day. (A) Representative gating strategy of the percentage of γδ and αβ T cells in ingoing CD3$^+$ T cell population and in CD3$^+$ T cells engrafting the xSCC 14 days post injection. (B) Summary graph showing γδ/αβ T cell ratio in CD3$^+$ T cells pre-transfer and in xSCC 2 days (n=7 mice, pool of 2 independent experiments), 7 days (n=12, pool of 4 independent experiments) and 14 days (n=12, pool of 2 independent experiments) post-transfer. Each symbol represents a γδ T cell donor. Data are shown as mean ± SD.

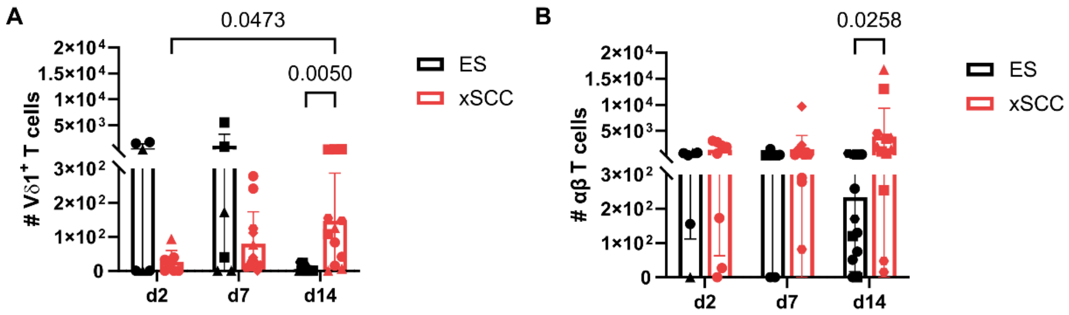

Figure S7. Tissue-dependent persistence of transferred Vδ1^{+} γδ and αβ T cells. 10x10^{6} human skin-derived T cells, containing approximately 7% of Vδ1^{+} γδ T cells and 93% of αβ T cells, were injected intravenously into NSG mice carrying a xSCC of a volume ranging from 100 to 200 mm^{3}. Each mouse was injected intraperitoneally with recombinant IL-2 and IL-15 daily until the harvest day. (**A,B**) Graphical summary of the absolute numbers of Vδ1^{+} γδ T cells (A) or αβ T cells (B) engrafting ES (black bar) and xSCC (red bar) 2 days (pool of 2 independent experiments), 7 days (pool of 4 independent experiments) and 14 days (pool of 2 independent experiments) post- transfer, normalized to tissue weight (grams). Each data point represents one mouse, and each symbol represents one skin donor. Data in bar graphs are shown as mean ± SD. Statistical significance was determined using two-way ANOVA with Šídák's multiple comparisons test.

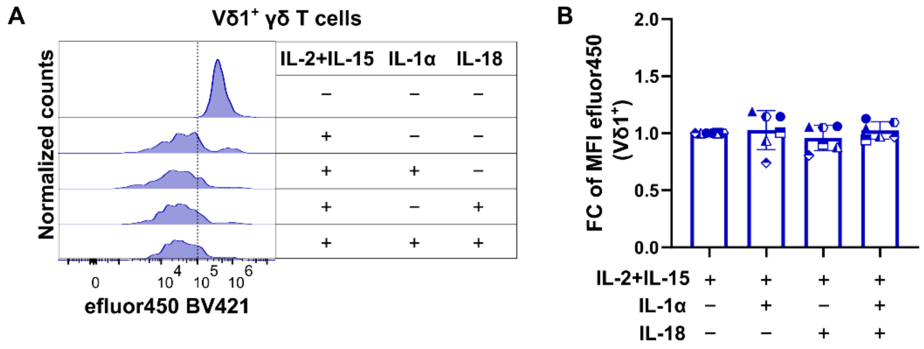

Figure S8. Effect of IL-1α and IL-18 on Vδ1^{+} γδ T cell proliferation in the absence of anti-CD3 stimulation. eFluor450-labelled γδ T cells were cultured under basal conditions (unstimulated or with IL-2 (100 IU/mL) and IL-15 (20 ng/mL)) or stimulated with IL-1α and/or IL-18 (9 ng/mL) for 6 days. Proliferation was assessed by the mean fluorescence intensity (MFI) of eFluor450 in Vδ1^{+} by flow cytometry. (**A**) The representative histograms show the eFluor450 dilution in Vδ1^{+} γδ T cells in the different conditions. Cell counts were normalized to unit area. (**B**) Bar graphs show the fold change of efluor450 MFI of Vδ1^{+} treated with IL-1α and IL-18 relative to IL-2 + IL-15 condition. Mean of n=6 skin donors. Data are shown as mean ± SD.

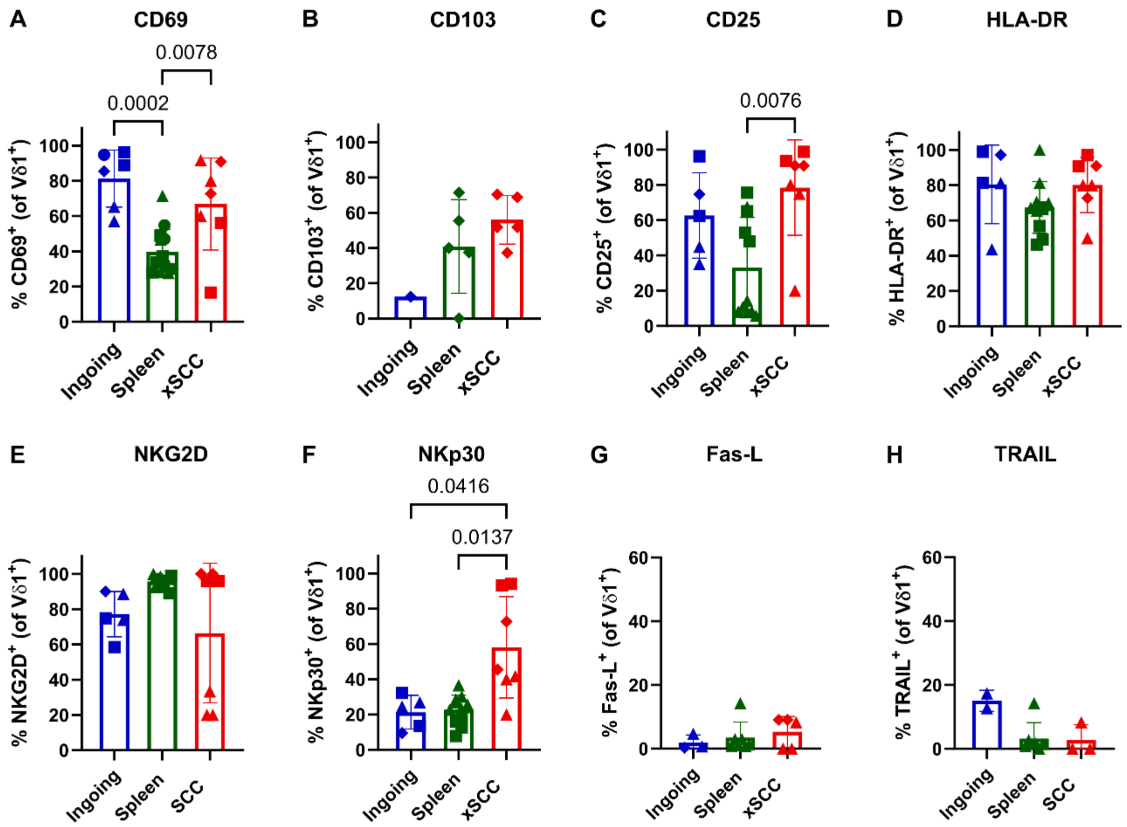

Figure S9. Phenotypic characterization of Vδ1$^+$ $\gamma\delta$ T cells isolated from xSCC tissues 7 days post-transfer. (A-H) Graphical summary of the percentage of: (A) CD69, (B) CD103, (C) CD25, (D) HLA-DR, (E) NKG2D, (F) NKp30, (G) Fas-L, and (H) TRAIL positive Vδ1$^+$ $\gamma\delta$ T cells before injection and isolated from spleen and xSCC. (A) n=9-15 mice per group, pool of 5 independent experiments. (B) n=5 mice per group, one experiment. Data were analysed using Ordinary one-way ANOVA with Tukey´s multiple comparisons test. (C-F) n=7-11 mice per group, pool of 5 independent experiments; (G) n=5-7 mice per group, pool of 3 independent experiments; (H) n=3-7 mice per group, pool of 2 independent experiments. Data were analysed using Kruskal-Wallis test with Dunn´s multiple comparisons test. Each symbol represents one skin donor. Data are shown as mean ± SD.

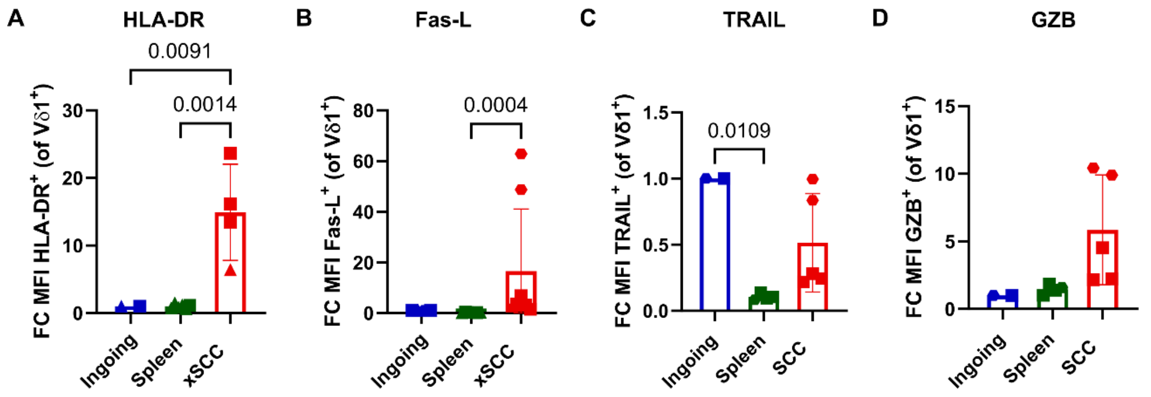

Figure S10. Vδ1$^+$ γδ T cell marker expression in spleen and xSCC relative to ingoing cells. (A-D) Graphical summary showing fold change (FC) of median fluorescence intensity (MFI) of Vδ1$^+$ γδ T cells for: (A) HLA-DR, (B) Fas-L, (C) TRAIL and (D) granzyme B (GZB) in spleen and xSCC relative to the corresponding ingoing Vδ1$^+$ population. Fold change (FC) was calculated for each donor as MFI in spleen or xSCC divided by MFI of the corresponding ingoing Vδ1$^+$ γδ T cells. (A) n=4-6 mice per group, one experiment. (B) n=6-8 mice per group, pool of 2 independent experiments. (C,D) n=4-5 mice per group, one experiment. (A,C) Data were analysed using Ordinary one-way ANOVA with Tukey´s multiple comparisons test. (B) Data were analysed using Kruskal-Wallis test with Dunn´s multiple comparisons test. Each symbol represents one skin donor. Data are shown as mean ± SD.

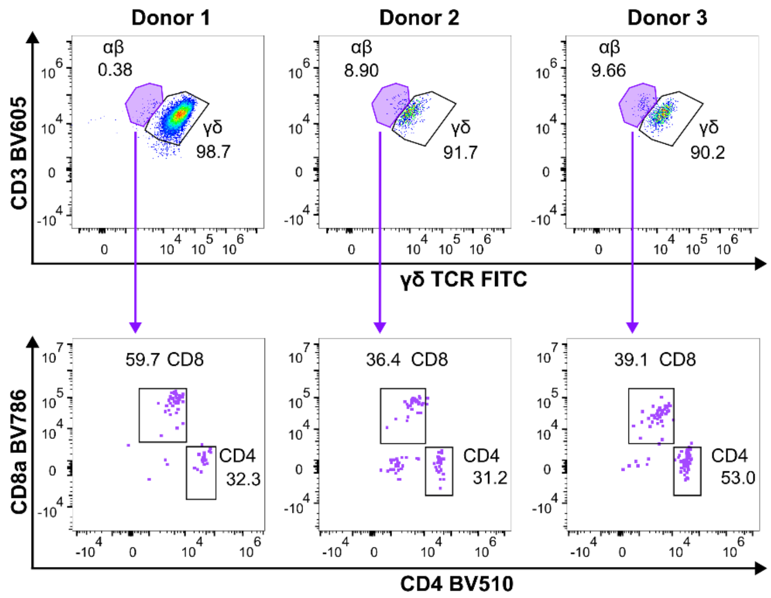

Figure S11. Purity of skin resident γδ T cells before adoptive transfer. γδ T cells from three skin donors were expanded *in vitro* with a proprietary recombinant human cytokine cocktail and isolated via positive selection by magnetic cell sorting. Top graph is showing the flow cytometry analysis of the proportion of γδ and αβ T cells of the total $CD3^+$ T cells after isolation. αβ T cells were further analysed for the expression of CD4 and CD8α markers (bottom graph).

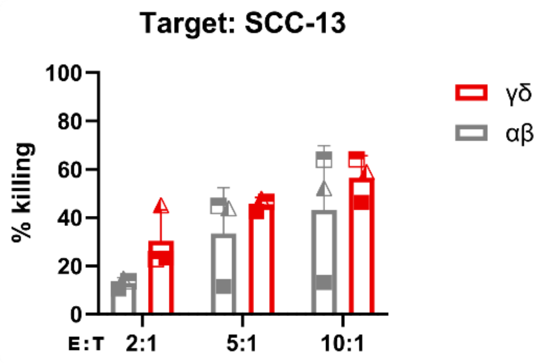

Figure S12. ***In vitro*** **cytotoxic activity of cutaneous γδ T cells and αβ T cells against SCC cells.** γδ and αβ T cells from three skin donors were expanded *in vitro* with a proprietary recombinant human cytokine cocktail and isolated via negative selection by magnetic cell sorting. SCC-13 cells expressing the luminescent reporter AkaLuc were co-cultured with purified (≥ 90%) γδ or αβ T cells at effector-to-target (E:T) ratios of 2:1, 5:1, and 10:1. After 22 hours, luminescent signal was measured using a TECAN plate reader as a readout for tumor viability. % killing was calculated relative to the luminescent signal of the target-only controls (i.e. 0:1). All conditions were performed in n=4 technical replicates and n=3 biological replicates.

Table S1: List of anti-human antibodies used for flow cytometry.

Antibody	Fluorochrome	Source	Identifier (Catalog number; RRID)
TCRγ/δ	FITC	Miltenyi	#130-113-511; AB_2733697
TCR Vδ1	FITC	Miltenyi	#130-118-362; AB_2751495
CD69	PerCP/Cyanine5.5	BioLegend	#310926; AB_2074956
CD45RA	PerCP/Cyanine5.5	BioLegend	#304122; AB_893357
TCR γ/δ	PE	Miltenyi	#130-114-038; AB_2751187
TCR Vδ1	PE	Miltenyi	#130-120-440; AB_2752099
CD218a (IL-18Rα)	PE	BioLegend	#313808; AB_345314
CD337 (NKp30)	PE/Dazzle 594	BioLegend	#325231; AB_2814184
CD3	PE/Cyanine5	BioLegend	#300410; AB_314064
CD314 (NKG2D)	PE-Vio770	Miltenyi	#130-111-646; AB_2657370
CD182 (CXCR2)	PE-Vio770	Miltenyi	#130-130-124; AB_2928293
CD103 (Integrin αE)	PE/Cyanine7	BioLegend	#350212; AB_2561599
CD45	APC	eBioscience	#17-0459-42; AB_10667894
CD178 (Fas-L)	APC	Miltenyi	#130-118-088; AB_2733348
TCR Vδ1	APC	Miltenyi	#130-118-968; AB_2733451
CD181 (CXCR1)	APC	Miltenyi	#130-115-880; AB_2727235
HLA-DR	Alexa Fluor700	BioLegend	#307626; AB_493771
CD4	Alexa Fluor700	eBioscience	#56-0049-42; AB_11219085
CD27	Alexa Fluor700	BioLegend	#356415; AB_2562515
CD25	Brilliant Violet 421	BD Horizon	#562442; AB_11154578
CD3	Brilliant Violet 421	BioLegend	#300434; AB_10962690

IL-1RAcP	Vio Bright V423	Miltenyi	#130-131-318; AB_2928712
CD45	Brilliant Violet 510	BioLegend	#304036; AB_2561940
CD45RA	Brilliant Violet 510	BioLegend	#304142; AB_2561947
Granzyme B	Brilliant Violet 510	BD Horizon	#563388; AB_2738174
CD183 (CXCR3)	Brilliant Violet 510	BioLegend	#353726; AB_2563642
CD69	Brilliant Violet 605	BioLegend	#310938; AB_2562307
CD253 (TRAIL)	Brilliant Violet 605	BD OptiBuild	#743720; AB_2741696
HLA-DR	Brilliant Violet 605	BioLegend	#307640; AB_2561913
CD27	Brilliant Violet 785	BioLegend	#302832; AB_2562674
CD45	Brilliant Violet 785	BioLegend	#304048; AB_2563129
CD196 (CCR6)	Brilliant Violet 785	BioLegend	#353422; AB_2563660
CD8a	Brilliant Violet 785	BioLegend	#301046; AB_2563264
